# Supplementary figures and images for: Re-evaluation of a Tn5::gacA mutant of Pseudomonas syringae pv. tomato DC3000 uncovers roles for uvrC and anmK in promoting virulence
Source: PLoS One. 2019 Oct 10;14(10):e0223637. doi: 10.1371/journal.pone.0223637 (PMC6786584; doi:10.1371/journal.pone.0223637)

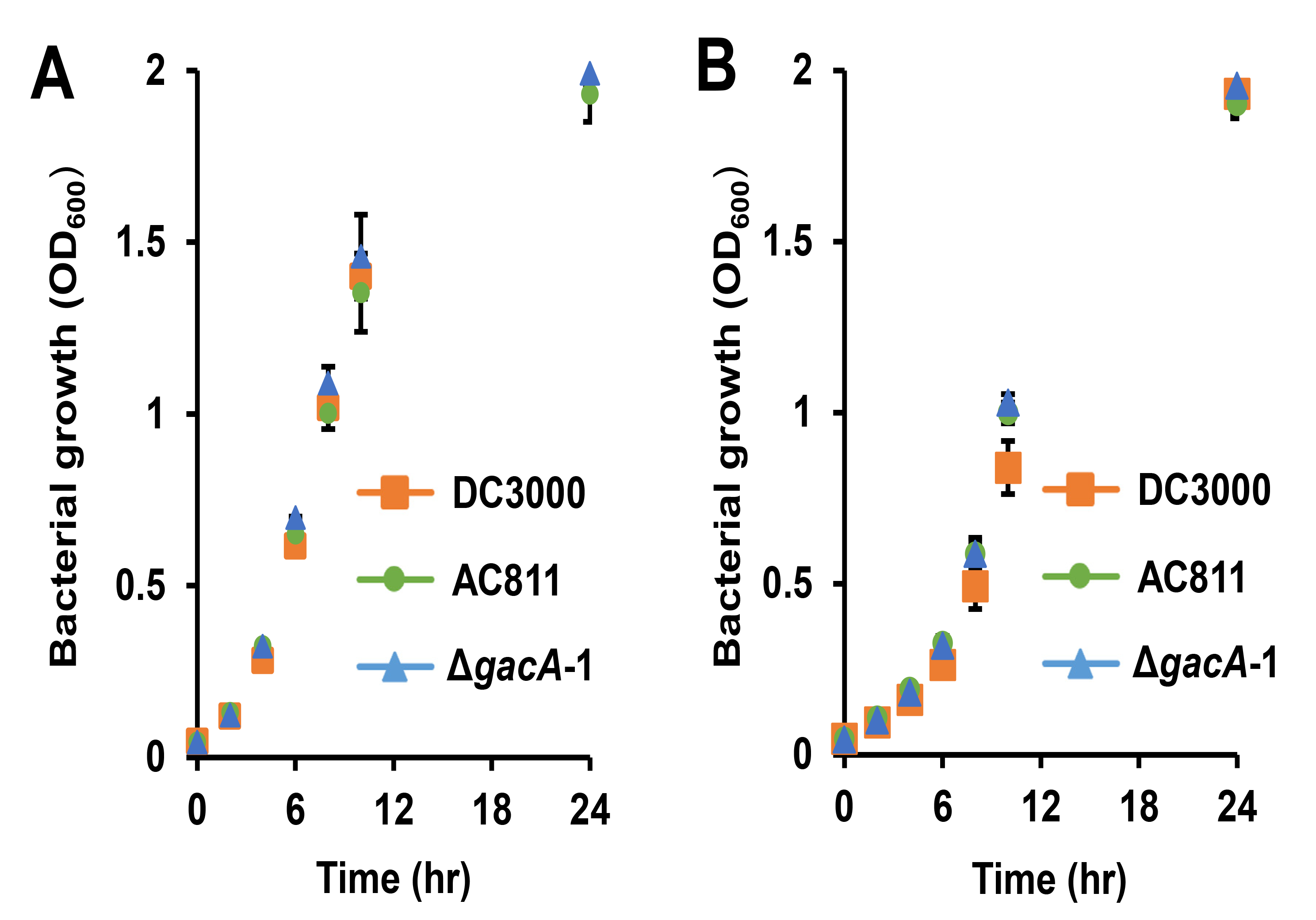

Supplement: S1 Fig — Time-course of bacterial growth at 28°C. DC3000, AC811 and ΔgacA-1 were inoculated at OD600 = 0.05 into KB broth and incubated at (A) 28°C or (B) 21°C with shaking. Graphed are means ± SE of OD600, n = 3. Data are pooled from three independent experiments. (TIF) [file pone.0223637.s001.tif]

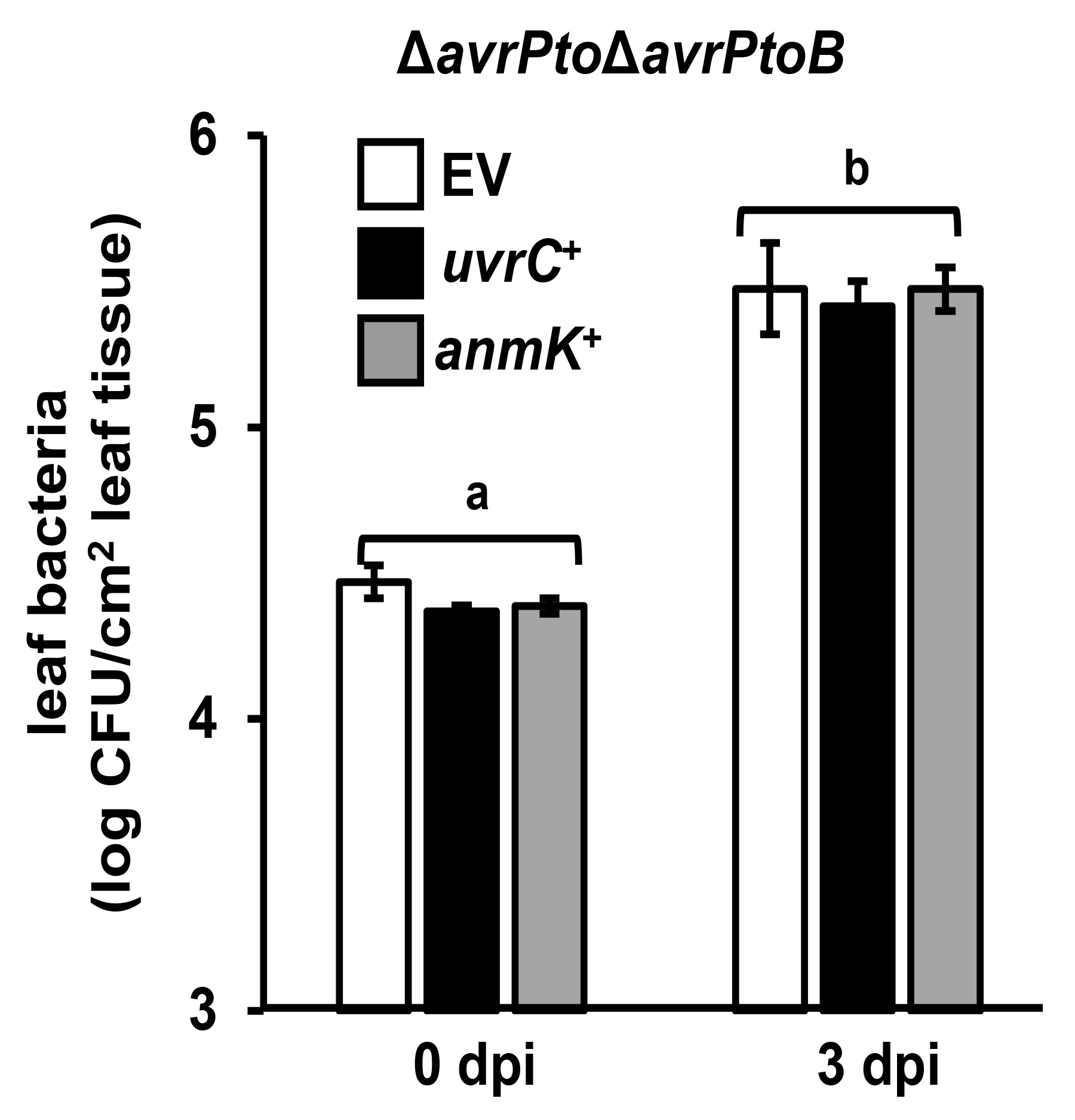

Supplement: S2 Fig — Arabidopsis leaves were syringe-infiltrated with DC3000 ΔavrPtoΔavrPtoB carrying empty pME6010 (EV), pME6010::uvrC, or pME6010::anmK as indicated. Abbreviation dpi is days post-infection. Graphed are means ± SE from data pooled from two independent experiments; n = 6. (TIF) [file pone.0223637.s002.tif]

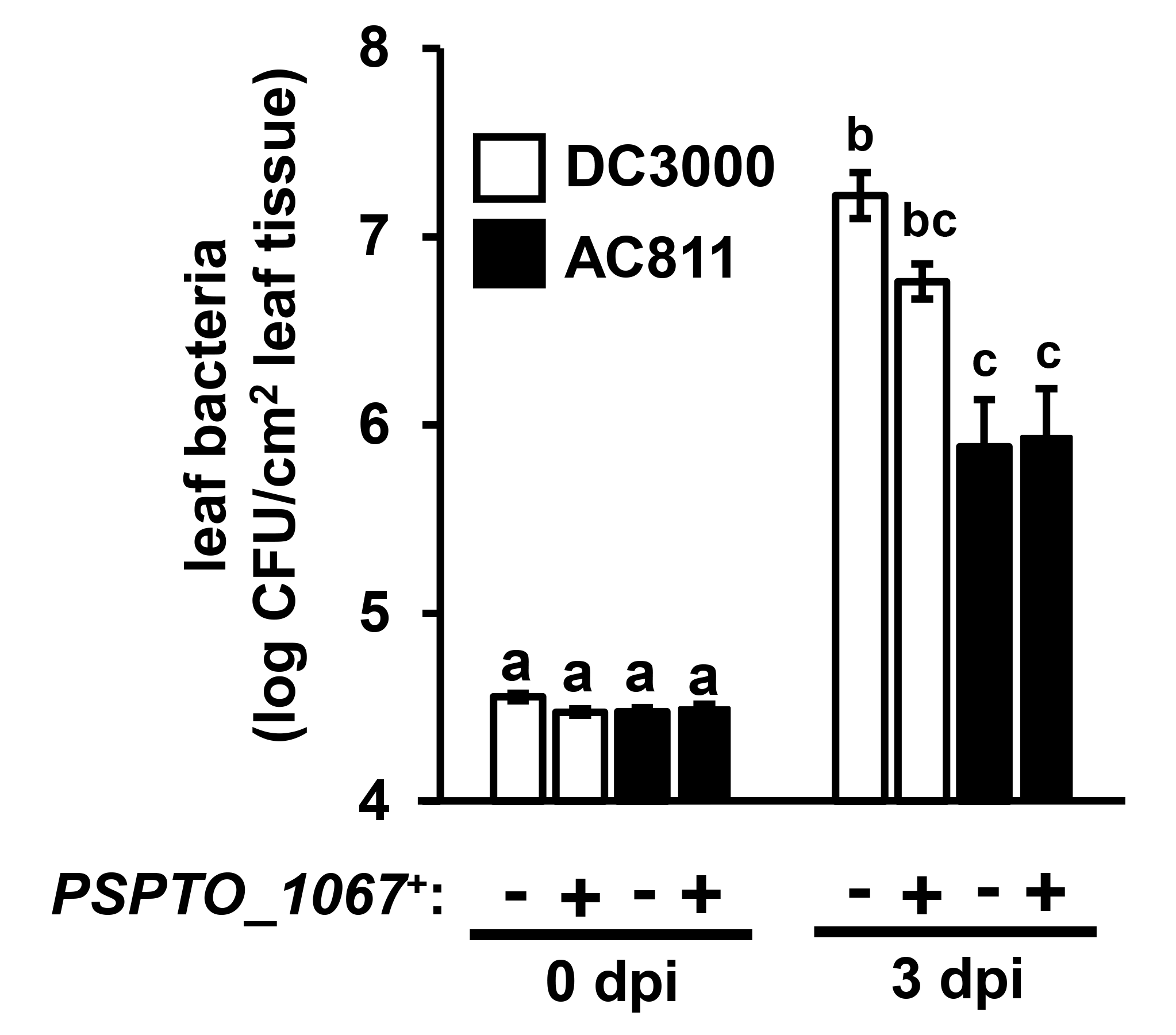

Supplement: S3 Fig — Arabidopsis leaves were syringe infiltrated with DC3000 and AC811 carrying either pME6010 empty vector or pME6010::PSPTO_1067 plasmid. Graphed are means ± SE of bacterial growth as determined by serial dilution plating, n = 3. Small-case letters denote statistical groups determined by ANOVA with multiple pairwise t-test comparisons and Tukey’s post-hoc HSD analysis, p < 0.05. Data are representative of two independent experiments. (TIF) [file pone.0223637.s003.tif]

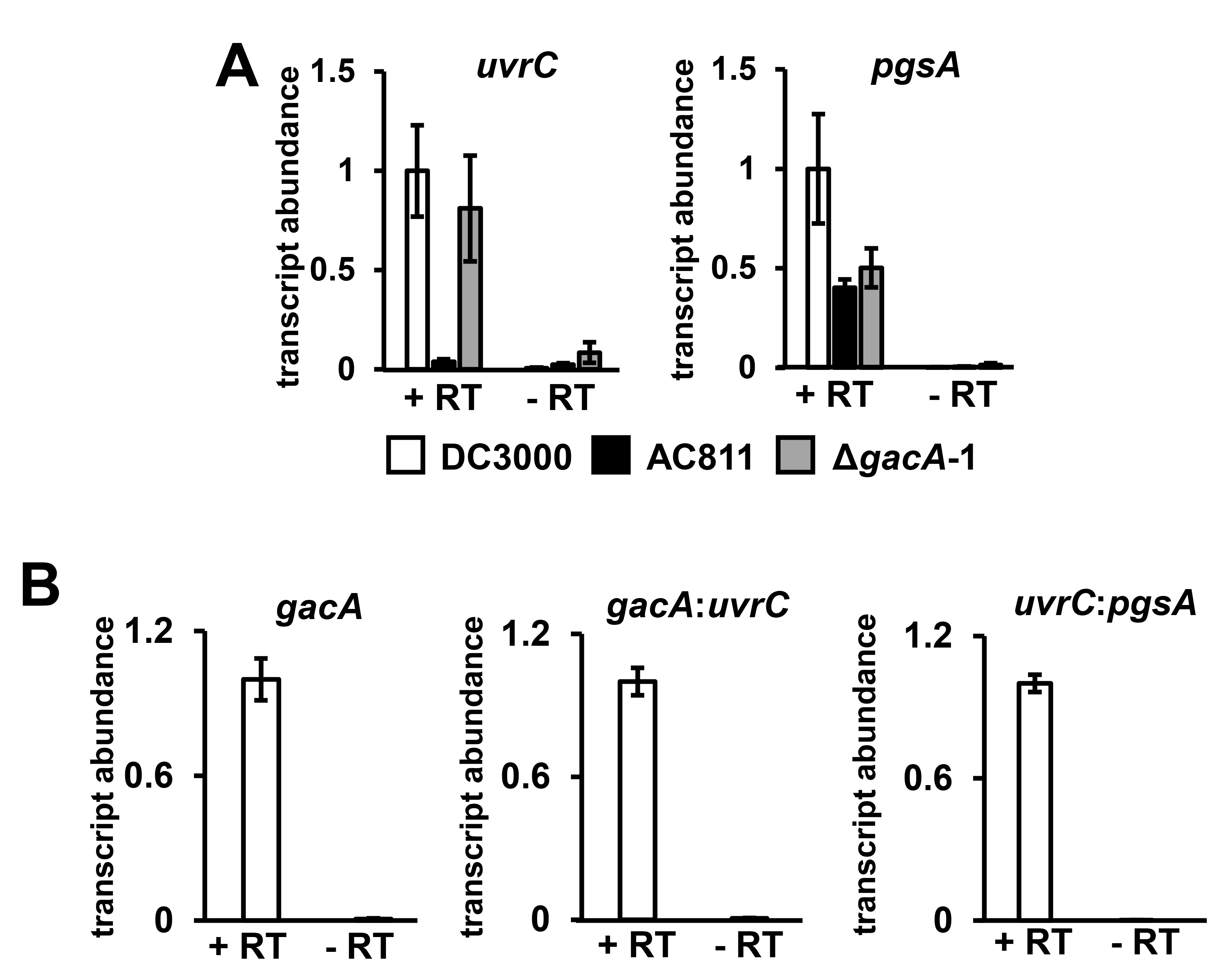

Supplement: S4 Fig — (A) RNA was extracted from DC3000, AC811, and ΔgacA-1, and cDNA synthesis performed with (+RT) or without reverse transcriptase (-RT). Shown is quantitative RT-PCR analysis of gacA, uvrC, and pgsA transcripts in these samples. Graphed are means ± SE of uvrC and pgsA transcript abundance normalized to gyrA from corresponding +RT samples and calculated relative to transcript levels measured in DC3000 (+RT). Data are pooled from two independent experiments with two technical replicates each; n = 4. Data for +RT samples are the same as shown in Fig 2B. (B) Transcript abundance of gacA, gacA:uvrC, and uvrC:pgsA junctions from DC3000 cDNA synthesized with (+RT) or without (-RT) reverse transcriptase. Data are pooled from two independent experiments with two technical replicates each; n = 4. (TIF) [file pone.0223637.s004.tif]

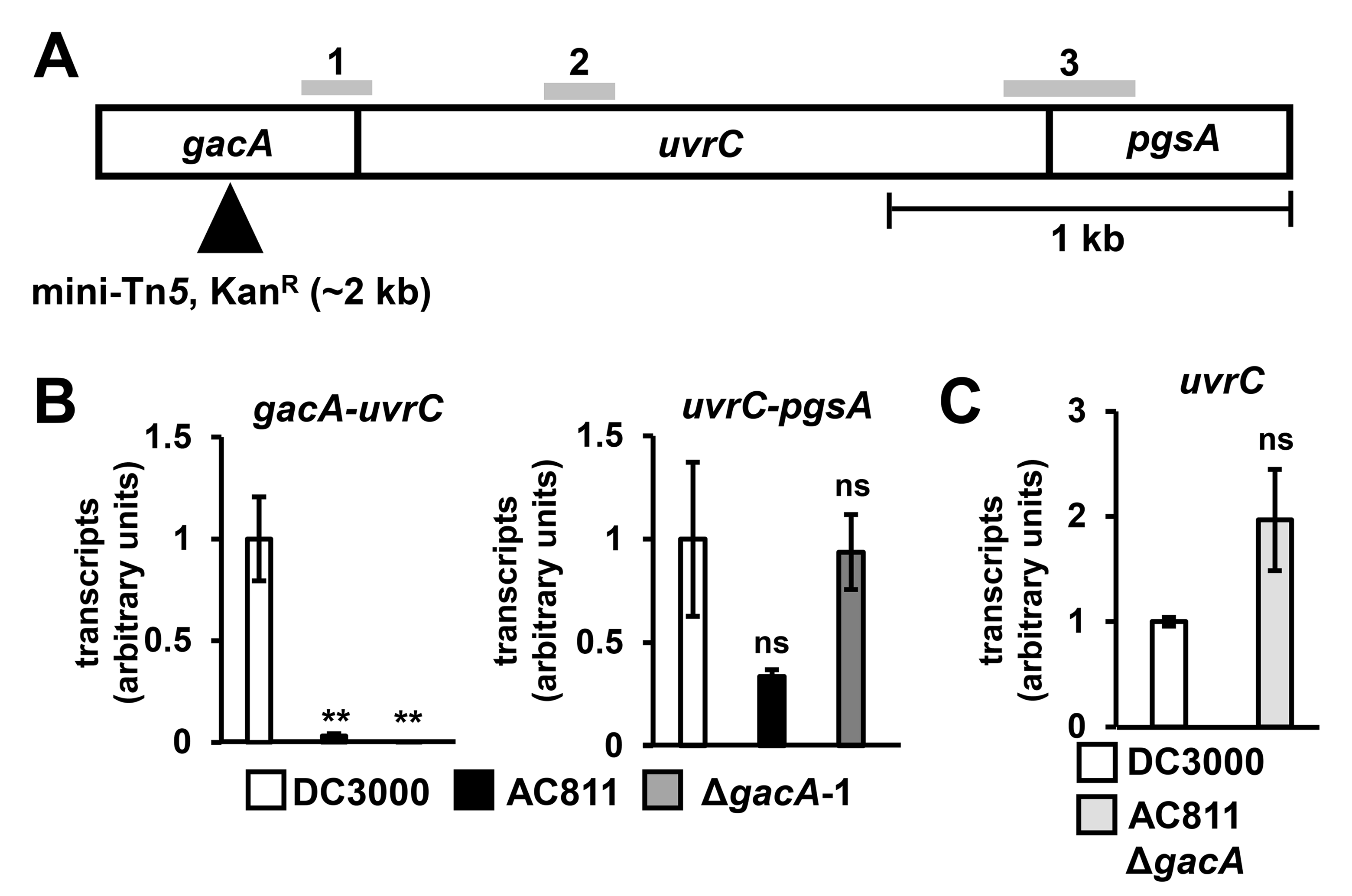

Supplement: S5 Fig — (A) Schematic of the predicted gacA-uvrC-pgsA operon in DC3000, with vertical bars indicating predicted translation start sites of uvrC and pgsA. Shading and numbering indicates regions targeted by qRT-PCR, with amplicons designated as follows: (1) gacA:uvrC; (2) uvrC; (3) uvrC:pgsA. (B) Abundance of uvrC transcripts was assessed by qRT-PCR using a gyrA reference gene as previously described. Values are normalized to DC3000. Graphed are means ± SE from data pooled across two independent experiments with two technical replicates each; n = 4. ns = no significant difference based on t-test (p > 0.05). (C) qRT-PCR analysis of transcripts containing gacA-uvrC or uvrC-pgsA junctions. Graphed are means ± SE from data pooled across two independent experiments with two technical replicates each; n = 4. **p < 0.01; ns = no significant difference (p > 0.05). (TIF) [file pone.0223637.s005.tif]

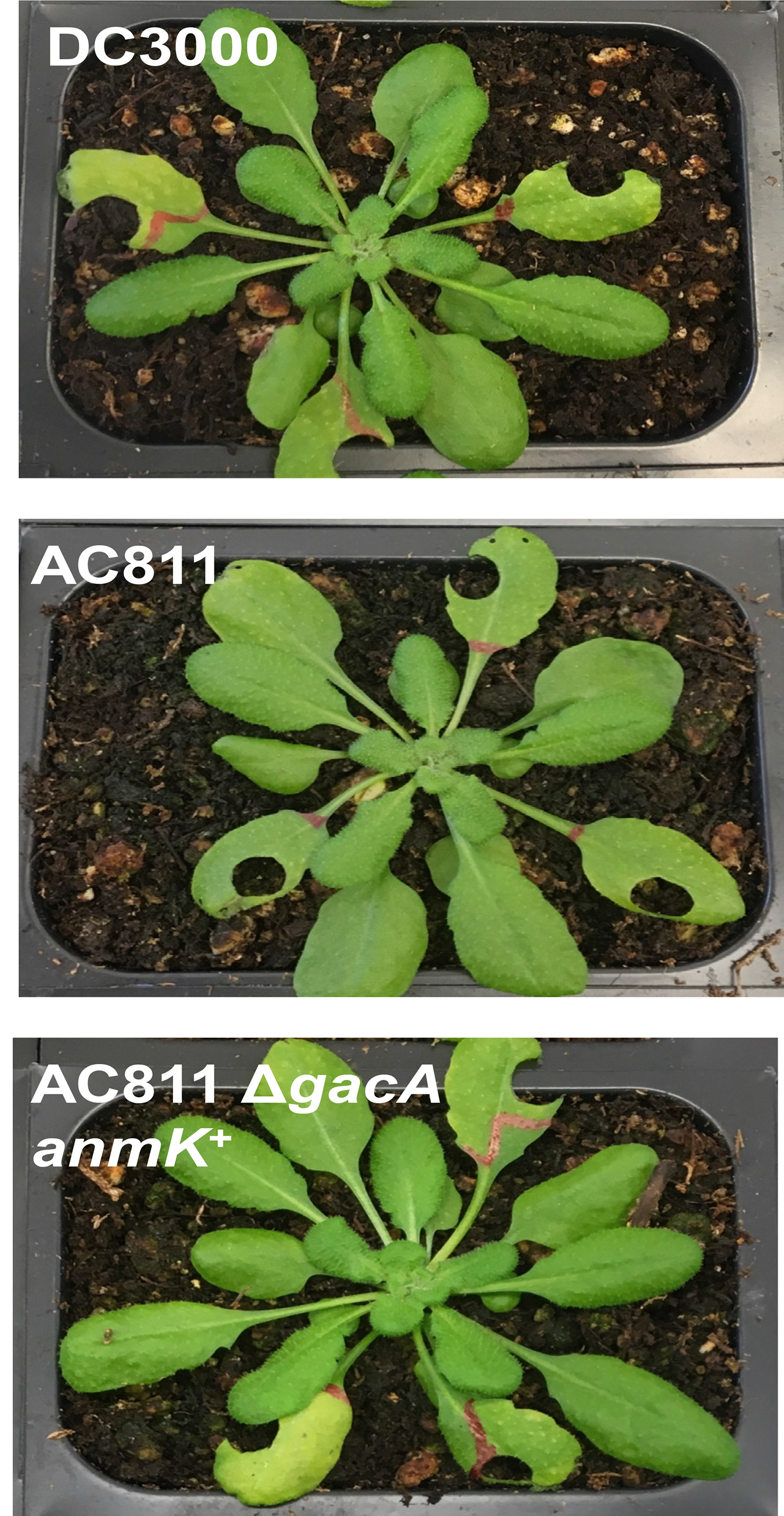

Supplement: S6 Fig — Arabidopsis leaves were syringe-infiltrated with DC3000, AC811, or AC811 ΔgacA carrying pME6010::anmK. Infected plants were photographed at 3 days post-infection (dpi). Circular punches on infected leaves are from sampling for bacterial cfu enumeration. Images are representative of symptoms observed in three independent experiments. (TIF) [file pone.0223637.s006.tif]
